# E‐learning and health inequality aversion: A questionnaire experiment

**DOI:** 10.1002/hec.3799

**Published:** 2018-07-22

**Authors:** Richard Cookson, Shehzad Ali, Aki Tsuchiya, Miqdad Asaria

**Affiliations:** ^1^ Centre for Health Economics University of York York UK; ^2^ Centre for Health Economics and Department of Health Sciences University of York York UK; ^3^ School of Health and Related Research (ScHARR), and Department of Economics University of Sheffield Sheffield UK; ^4^ LSE Health London School of Economics and Political Science (LSE) London UK

**Keywords:** distributional cost‐effectiveness analysis, empirical ethics, empirical social choice, health inequality, inequality aversion

## Abstract

In principle, questionnaire data on public views about hypothetical trade‐offs between improving total health and reducing health inequality can provide useful normative health inequality aversion parameter benchmarks for policymakers faced with real trade‐offs of this kind. However, trade‐off questions can be hard to understand, and one standard type of question finds that a high proportion of respondents—sometimes a majority—appear to give exclusive priority to reducing health inequality. We developed and tested two e‐learning interventions designed to help respondents understand this question more completely. The interventions were a video animation, exposing respondents to rival points of view, and a spreadsheet‐based questionnaire that provided feedback on implied trade‐offs. We found large effects of both interventions in reducing the proportion of respondents giving exclusive priority to reducing health inequality, though the median responses still implied a high degree of health inequality aversion and—unlike the video—the spreadsheet‐based intervention introduced a substantial new minority of non‐egalitarian responses. E‐learning may introduce as well as avoid biases but merits further research and may be useful in other questionnaire studies involving trade‐offs between conflicting values.

## 1 INTRODUCTION

A large body of evidence from economics, psychology, sociology, anthropology, and neuroscience suggests that people are averse to inequality in outcome distributions (Dawes, Fowler, Johnson, McElreath, & Smirnov, 2007; Tricomi, Rangel, Camerer, & O'Doherty, 2010). People seem averse not only to inequality relating to their own outcome—what has been dubbed “self‐centred” or “comparative” inequality aversion (Clark & D'Ambrosio, 2015; Fehr & Schmidt, 1999)—but also to inequality within society as a whole, or “normative” inequality aversion (Alesina, Giuliano, Bisin, & Benhabib, 2011; Alesina & La Ferrara, 2005). In recent decades, the interdisciplinary field of empirical social choice has emerged to study normative inequality aversion and social attitudes towards fairness and distributive justice more generally, with seminal contributions from economists as well as philosophers, sociologists, and psychologists (Amiel & Cowell, 1999; Gaertner & Schokkaert, 2012; Konow, 2003; Yaari & Bar‐Hillel, 1984).

Information about comparative inequality aversion can help to predict individual behavior, such as costly punishment and rewarding of others to avoid unequal outcomes (Fehr & Fischbacher, 2003). This is often studied in economic laboratory experiments with financial incentives, to help ensure that the subjects behave in a similar way to individuals engaging in real economic transactions in the field (Binmore, 1999; Binmore & Shaked, 2010). By contrast, studies of normative inequality aversion use questionnaire experiments without financial incentives, because the primary aim is to understand social attitudes about fairness and justice rather than to predict individual behavior. Information about social attitudes can help us to understand public policy making (Alesina & Angeletos, 2005), to challenge and refine theories of justice (Miller, 1992), and to recognize social influences on our own value judgments (Alesina & Giuliano, 2009).

One strand of this literature in the health field has investigated health inequality aversion, that is, social attitudes towards trade‐offs between improving sum total health and reducing socioeconomic inequality in health (Abasolo & Tsuchiya, 2004; Ali, Tsuchiya, Asaria, & Cookson, 2017; Cropper, Krupnick, & Raich, 2016; Dolan & Tsuchiya, 2011; Edlin, Tsuchiya, & Dolan, 2012; Robson, Asaria, Cookson, Tsuchiya, & Ali, 2016), in the context of a wider literature on equity and the economic evaluation of health programs (Baker et al., 2010; Cookson, Griffin, & Nord, 2014; Cookson et al., 2017; Donaldson et al., 2011; Lancsar, Wildman, Donaldson, Ryan, & Baker, 2011). In these studies, health inequality aversion can be quantified using a parameter in a social welfare function. For example, in one standard formulation of the Atkinson (1970) welfare function, a parameter value of zero represents no concern for health inequality (what we might call a “utilitarian” view), and increasingly positive numbers represent increasing priority to less healthy people—with the limiting case of exclusive priority to the least healthy person or group as the parameter approaches infinity (what we might call a Rawlsian “maximin” view).

Estimates of health inequality aversion can be used as normative benchmarks to help guide health policymakers who wish to ensure that the values underpinning the decisions they make are reasonably well aligned with the values of the general population. For example, policymakers might face a choice between two ways of increasing uptake of a publicly funded program of screening for bowel cancer inequality (Asaria, Griffin, Cookson, Whyte, & Tappenden, 2015): a standard universal reminder campaign aimed at the entire eligible population or a targeted campaign focusing marketing resources more intensively on disadvantaged populations with relatively poor health and low screening uptake. The standard approach might deliver a greater sum total net health benefit, insofar as advantaged populations are more responsive to low‐cost reminder messages, whereas the targeted approach might reduce health inequality (Asaria et al., 2015). The policymaker then would need to make a social value judgment about this trade‐off. To help inform this judgment, it is not enough to know simply whether the public is averse to socioeconomic health inequality. The policymaker needs to know: how averse?

All public opinion surveys are vulnerable to framing effects and other cognitive biases due to people's limited ability to process information (Kahneman, 2011; Simon, 1982), and empirical social choice surveys are no different (Hurley, Buckley, Cuff, Giacomini, & Cameron, 2011). Studies of health inequality aversion that use one standard questionnaire approach (Dolan & Tsuchiya, 2011; Shaw et al., 2001) typically find that a high proportion of respondents—sometimes even a majority—are so strictly “pro‐egalitarian” that they appear to give exclusively priority to reducing health inequality and unwilling to make trade‐offs with improving total health (Abasolo & Tsuchiya, 2004, 2013; Ali et al., 2017). These studies use a simple “matching” question that compares health gains for two groups with different baseline levels of quality‐adjusted life expectancy at birth—for example, a “rich” group and a “poor” group—in an iterative sequence of pairwise choices with varying levels of health inequality and total health. In all such studies, a high proportion of respondents either (a) give exclusive priority to improving the health of the poor group, even if this means lower health for the poor group, or (b) reduce inequality by “leveling‐down” the health of the rich group without increasing the health of the poor group, or (c) reduce inequality by leveling‐down the health of both groups. Because these choices all imply that social welfare can be improved by reduction in health, they violate the monotonicity condition that social welfare is a non‐decreasing function of individual good (in this context, health). In two studies in Spain, more than 50% were willing to “level down” the health of both groups—that is, case (c) above (Abasolo & Tsuchiya, 2004, 2013). These kinds of strict pro‐egalitarian views that appear unwilling to make trade‐offs are “off the scale” of inequality aversion as quantified using standard monotonic social welfare functions: An Atkinson parameter tending to infinity can represent the goal of maximizing the health of the worst off—that is, case (a) above—but cannot prescribe leveling‐down the health of either group—thatis, cases (b) and (c). Such findings also have uncomfortable implications for public policymakers, who generally dislike leveling‐down in the domain of health (Dutta, 2007). Furthermore, although it is debatable whether degrees of inequality aversion are (or ought to be) comparable across domains of well‐being, these findings do not cohere with wider evidence about social attitudes, such as studies of normative income inequality aversion, which typically find median inequality aversion parameters of between 0.5 and 3 (Clark & D'Ambrosio, 2015).

One response to the finding that a high proportion of respondents appear to favor leveling‐down of health has been to assume that this finding reflects true preferences and to develop new welfare functions that accommodate violation of monotonicity beyond certain levels of inequality (Abasolo & Tsuchiya, 2004). An alternative response, pursued in this study, is to adopt the working hypothesis that this standard questionnaire approach is vulnerable to a “pro‐strict‐egalitarian” cognitive bias favoring exclusive priority to reducing health inequality and unwillingness to make trade‐offs and to explore ways of mitigating this potential bias. Specifically, some people may give apparently extreme responses that do not accurately reflect their social attitudes, because they think about the question in an incomplete way that ignores the trade‐off with total health and focuses only on reducing health inequality as if this were a “sacred value” that cannot be traded‐off against other values (Tetlock, 2003; Tetlock, Kristel, Elson, Green, & Lerner, 2000). Incomplete thinking might occur because respondents have limited expertise in thinking about abstract questions involving trade‐offs between competing values and consequently only a limited understanding of the question being asked.

This study aims to mitigate this potential bias by developing and testing two e‐learning educational interventions designed to help respondents think about the question in a more complete manner by gaining greater expertise and understanding about the nature of the trade‐off and the implications of their responses. In economic laboratory experiments, the main learning mechanism is trial‐and‐error learning from experience of repeated decisions (Binmore, 1999; Binmore & Shaked, 2010). Trial‐and‐error learning is hard to implement in empirical social choice studies, however, because simply repeating social attitude questions is unlikely to facilitate learning in the absence of any financial payoff or other feedback. So we need to explore different learning mechanisms. We define e‐learning as education conducted via electronic media. An advantage of e‐learning over traditional face‐to‐face education is that it can be administered in a consistent and auditable manner. Each subject receives the same e‐learning intervention, and the content is open to external scrutiny so that third parties can assess how far it facilitates understanding rather than merely introducing new forms of bias—in other words, how far it represents education versus propaganda. The first e‐learning intervention used in this study was a video animation debate between characters arguing for different choices based on different ethical principles, including both maximizing total health and equalizing health. The second was a spreadsheet‐based interactive version of the questionnaire providing feedback on the implications of alternative choices for both total health and health inequality. We randomized respondents to receive either a standard “paper” questionnaire or the spreadsheet‐based “interactive” questionnaire and asked them to complete the survey before and after the “video” intervention.

We found that both e‐learning interventions had substantial effects. Both the video intervention and the interactive questionnaire substantially reduced the proportion of strict egalitarian responses compared with the standard paper questionnaire. The interactive questionnaire—but not the video—also resulted in substantially more respondents expressing strict non‐egalitarian views. In both cases, however, the median respondent still had a high degree of health inequality aversion. The median Atkinson inequality aversion parameter was 5.4 in the interactive questionnaire group and 10.9 in the post‐video paper questionnaire group, implying that a marginal health gain was still valued much more highly for the “poorest fifth” than the “richest fifth,” by multiples of 2.6 and 7.0, respectively.

## 2 METHODS

### 2.1 Questionnaire

The questionnaire instrument used in this study (see Appendix A) is a two‐group health outcome matching question adapted from Abasolo and Tsuchiya (2013) and Ali et al. (2017). It starts by presenting the current level of inequality in health across two socioeconomic groups in England. In this study, the two groups used are “the richest fifth,” who on average live 74 years in full health, and “the poorest fifth,” who on average live 62 years in full health (Love‐Koh, Asaria, Cookson, & Griffin, 2015). Both groups are made up of around 10 million individuals.

Respondents are then presented with a sequence of seven pairwise choices between two health programs, A and B, designed to iterate towards a matching point of indifference. In each pair Program A gives a 7‐year gain in life in full health to the richest fifth and a 3‐year gain in life in full health to the poorest fifth, that is, an increase in health inequality. In the first pair, Program B gives a 3‐year gain to the richest fifth and an 8‐year gain to the poorest fifth. Program B thus starts out by offering a larger gain in total health and a reduction in health inequality. In the subsequent six pairs, the health gain to the poorest in Program B decreases gradually from 8 to 2 years while everything else remains the same. Hence, Program B gradually offers a smaller reduction in health inequality (switching to an increase in health inequality in Pair 7) and a smaller gain in total health, whereas Program A remains the same. Appendix A reproduces the seven pairwise choices; the full questionnaire is available from the authors on request.

### 2.2 Response classification system

The questionnaire accommodates a broad spectrum of views about justice. Specifically, it allows us to distinguish five different principles of health justice, as explained below, which are labeled in Table 1 as “Pro‐rich,” “Health maximizer,” “Weighted prioritarian,” “Maximin,” and “Extreme egalitarian.” Respondents who prefer Program A in either of the first two choices are classified as Pro‐rich. These respondents can be thought of as “inequality seeking,” because they prefer a program that increases health inequality without increasing total health. Respondents are classified as Health maximizer if they are indifferent between the programs in the second choice—when total health is the same under both programs—but otherwise always choose the program delivering more total health.

**Table 1:** Response classification system

Category	Label	Response pattern (paper)^a^	Response range (interactive)^b^	Point of indifference^c^
1	Pro‐rich 1	AAAAAAA	>8	>8.0
2	Pro‐rich 2	=AAAAAA	8	8.0
3	Pro‐rich 3	BAAAAAA	7.1–7.9	7.5
4	Health maximizer	B=AAAAA	7.0	7.0
5	Weighted prioritarian 1	BBAAAAA	6.5–6.9	6.5
6	Weighted prioritarian 2	BB=AAAA	6.0–6.4	6.0
7	Weighted prioritarian 3	BBBAAAA	5.5–5.9	5.5
8	Weighted prioritarian 4	BBB=AAA	5.0–5.4	5.0
9	Weighted prioritarian 5	BBBBAAA	4.5–4.9	4.5
10	Weighted prioritarian 6	BBBB=AA	4.0–4.4	4.0
11	Weighted prioritarian 7	BBBBBAA	3.1–3.9	3.5
12	Maximin	BBBBB=A	3.0	3.0
13	Extreme egalitarian 1	BBBBBBA	2.5–2.9	2.5
14	Extreme egalitarian 2	BBBBBB=	2.0–2.4	2.0
15	Extreme egalitarian 3	BBBBBBB	<2.0	<2.0

a At each pair, respondents have three choices: Program A, Program B, or indifference. We represent these three choices using the Characters A, B, and =, respectively. So, for example, respondents who prefer Program A in all seven pairs are denoted (AAAAAA).

b To facilitate analytical and graphical comparisons with the paper questionnaire results, the continuous response scale was converted into a discrete scale by dividing it into ranges and interpreting the midpoint of the corresponding range as the discrete point where the respondent is indifferent between A and B.

c This is the number of years to the poorest fifth in Program B at the point where the respondent is indifferent between A and B. For those categories where the respondent switches directly from B to A, it is assumed to be at the midpoint of A and B.

Our third type is the “trader” or “weighted prioritarian.” The term weighted prioritarian means people who give priority to the worse off but not exclusive priority (Arneson, 2013): They still give some weight to gains for the better off. Weighted prioritarians will make trade‐offs between improving total health and reducing health inequality but will not violate monotonicity.

We classify a respondent as maximin if they choose the program that has the largest impact on improving the health of the poorest fifth regardless of any concern for total health. A maximin respondent will be indifferent in the sixth choice, in which the health of the poorest fifth is the same in both programs, whereas a weighted prioritarian will choose Program A. Finally, we classify a respondent as “extreme egalitarian” if they are willing to “level‐down” the health of either group in ways that violate monotonicity. An “extreme egalitarian 1” respondent is willing to sacrifice health gain in the richest group without increasing the health of the poorest group, and “extreme egalitarian 2 and 3” respondents are willing to sacrifice health gain in both groups if this will reduce health inequality. Collectively, we group together the last four labels—including maximin and the three subtypes of extreme egalitarian—under the umbrella label “strict egalitarian.”

In the paper questionnaire, unlike the interactive version, respondents can give apparently inconsistent response patterns that introduce a degree of ambiguity in classification. Our base case analysis includes such subjects whose responses appear to reflect a minor transient response error or imprecise preferences, with the ambiguity resolved by the rules described in Appendix B, though we conduct sensitivity analysis excluding these subjects.

#### 2.2.1 E‐learning interventions

Two screen shots of the interactive questionnaire are provided in Appendix C. The respondent is able to move the “slider” in the middle of the screen to explore the implications of different choices, before pressing the “done” button to make a choice. The top half of the page shows the initial screen where the slider is set to the top of the scale (corresponding to the “Pair 1” choice in the paper questionnaire), and the bottom half shows an example screen where the slider has been moved approximately half way down the scale (corresponding to the “Pair 5” choice in the paper questionnaire). A transcript of the video animation is in Appendix D and a screen shot in Appendix E. The full video and interactive questionnaire are publicly available via the University of York website (http://www.york.ac.uk/che/research/equity/economic_evaluation/publicviews/).

### 2.3 Data collection

As is common in questionnaire studies of social attitudes, we administered the questionnaires in a face‐to‐face setting following a discussion group “warm up” session beforehand where all participants in both intervention groups interacted, were given background information about the topic, and completed related attitude questions on their own.

Members of the public were invited to participate in one of two discussion group events, involving 30 people each. Recruitment was via advertisements in a monthly free local magazine (*Your Local Link*) distributed to all homes across York (35 postcode sectors) in January 2014. Those interested were asked to contact the research team by phone, post, or email with their name, contact details, age, sex, and postcode. A quota was set by four age groups and sex so that each age/sex group had a capacity for three to four participants. Due to our recruitment strategy in the local area, in which the University of York is a major employer, those with university academic or research jobs were excluded to avoid risk of over‐sampling of these groups or the participation of students or colleagues known to research team members.

Participants were randomized as they arrived for registration on the day, to complete either the paper or the spreadsheet‐based interactive questionnaire. Descriptive statistics are reported separately for the paper and interactive samples in terms of demographic variables and answers to political attitude questions.

Each event lasted 5 hr, and payments of £50 were offered to each participant. The tasks reported in this paper were undertaken in the morning session, from 9:30 to 11:45, with further tasks thereafter. The events were held at a location easily accessible by public transport, Heslington East Campus of the University of York, on Saturday April 26 and Saturday May 3, 2014.

Each event started with respondents having tea and coffee together in the waiting room. There was then a plenary warm up session, lasting about 30 min, starting with an introductory presentation by the lead investigator giving background information about health inequality and proceeding to the respondents completing a set of standard questions from the British Attitudes Survey about attitudes to the welfare state and income redistribution (Jowell & Witherspoon, 1985). One group then went to the computer laboratory to complete the interactive questionnaire, whereas the other group stayed behind to complete the paper questionnaire.

The computer laboratory session began with a presentation by one of the research team, taking respondents through the initial “tutorial” steps of the interactive questionnaire. Respondents then completed the interactive questionnaire individually, with facilitators on hand to answer clarification questions (including the research team and two PhD students). After this, respondents were shown the video and asked to complete a booklet on the video containing comprehension questions designed to encourage further reflection. Finally, respondents were asked to complete the interactive questionnaire a second time.

The paper questionnaire session began with respondents separating into three small groups of five or six, in separate rooms. Respondents answered the paper questionnaire individually, but discussion was allowed, and a facilitator was on hand to answer clarification questions. After completing their responses, respondents were brought back into the main room to watch the video and then complete the video booklet. Finally, respondents went back into their small groups to complete the questionnaire a second time.

Research ethics approval was obtained from the University of York Health Sciences Research Governance Committee.

### 2.4 Analysis

Our research design has four data collection modes: (a) pre‐video paper, (b) pre‐video interactive, (c) post‐video paper, and (d) post‐video interactive. Our two main comparisons are (a) versus (b), to test the effect of the interactive questionnaire compared with the standard paper questionnaire, and (a) versus (c), to test the effect of the video on the standard paper questionnaire. We are also interested in (b) versus (d), the effect of the video on the interactive questionnaire, and (c) versus (d), the difference between the paper and interactive questionnaires after exposure to the video. We first present descriptive statistics for the four treatments, showing the proportion of respondents classified under each of our five principles of health justice. More fine‐grained detail is presented for our four comparisons of interest using cumulative distributions across the 15 ordered response categories (from less to more egalitarian). We then present nonparametric tests of significance, including Wilcoxon rank‐sum tests of the null hypothesis of no difference in cumulative ranks and equality of proportions tests of the null hypothesis of no difference in the probability of a strict egalitarian response (maximin or extreme egalitarian) and the probability of a non‐egalitarian response (“pro‐rich” or “health maximizer”). Finally, we present regression results that allow for respondent covariates, based on the response model:

$$y_{\mathrm{it}}^{*}=\beta_{1}{\text{interactive}}_{\mathrm{it}}+\beta_{2}{\text{postvideo}}_{\mathrm{it}}+\beta_{3}{\text{interactive}}_{\mathrm{it}}\times {\text{postvideo}}_{\mathrm{it}}+\beta_{4}X_{i}X_{i}+\varepsilon_{\mathrm{it}},$$

where $y_{\mathrm{it}}^{*}$ represents the unobserved judgment of respondent *i* at task *t* and the explanatory variables consist of both main treatment effects (*interactive* and *post‐video*) and an interaction term (interactive * post‐video), with and without covariates ***X*** for respondent characteristics (age group, gender, and deprivation quintile group). We use random effects ordered probit to model the joint probability distribution for each of the five principles. We adjust standard errors to account for clustering of responses within subjects. The first two main treatment effect coefficients (*β*_1_ and *β*_2_) represent the two main comparisons of interest. We also present our two further comparisons of interest based on joint tests involving the interaction term, (*β*_3_).

## 3 RESULTS

### 3.1 The sample

One of the 60 subjects gave responses that appear to reflect a clear misunderstanding of the question and was excluded from the base case analysis. Five subjects gave responses that appear to reflect a transient response error or imprecise preferences with indifference spilling across two or more pairs, and these were reinterpreted and included in the sample as described in Appendix B. As Table 2 shows, the resulting samples in the paper and interactive treatment groups were similar in terms of social attitudes and neighborhood deprivation, though the paper group had a lower proportion of males and people aged 35–49.

**Table 2:** Sample characteristics

	Paper group (*N* = 29)		Interactive‐questionnaire group (*N* = 30)	
Characteristic	Statistic	*n*	Statistic	*n*
Male (%)	38%	11	47%	14
Age (%)				
18–34	31%	9	20%	6
35–49	7%	2	27%	8
50–64	38%	11	20%	6
65+	24%	7	33%	10
Deprivation quintile group^a^ (mean; 1 = *most deprived*; 5 = *most affluent*)	3.41	29	3.70	30
Social attitude statements^b^ (mean; 1 = *strongly agree*; 5 = *strongly disagree*)				
The creation of the welfare state is one of Britain's proudest achievements.	1.79	29	1.77	30
Government should redistribute income from the better‐off to those who are less well off.	3.03	29	3.10	30

a Deprivation quintiles were based on postcode of respondents which were matched to Index of Multiple Deprivation.

b 1 suggests most egalitarian and 5 suggests least egalitarian.

### 3.2 The distribution of responses by treatment group

**Figure 1:**
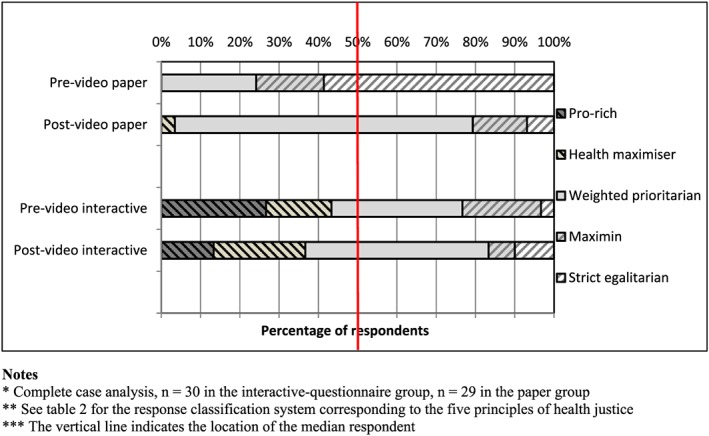
Inferred principles of health justice by question and sample design [Colour figure can be viewed at wileyonlinelibrary.com]

Figure 1 shows the distribution of responses across the five principles of health justice. Each stacked bar indicates the proportion of responses ranging from pro‐rich on the left to extreme egalitarian on the right. The median respondent in the pre‐video paper sample was extreme egalitarian, switching to weighted prioritarian post‐video. By contrast, in the interactive group, the median respondent was weighted prioritarian both pre‐ and post‐video. The proportion in the paper group expressing a strict egalitarian view (either maximin or extreme egalitarian) fell substantially from 75.9% pre‐video to 20.7% post‐video (*p* = 0.00). The proportion of the pre‐video interactive‐questionnaire group expressing a strict egalitarian view was 23.3%, which is substantially lower than the proportion in the pre‐video paper group (*p* = 0.00). On the other hand, although the proportion expressing an extremely non‐egalitarian view (i.e., pro‐rich) was zero in the paper group both before and after the video, it was significantly higher in the interactive‐questionnaire group (26.7% higher before, with *p* = 0.00, and 13.3% higher after, with *p* = 0.04). Additionally, the proportion of “health maximizers” was also higher in the interactive‐questionnaire group compared with the paper group (16.7% higher before video, with *p* = 0.02, and 19.9% higher after video, with *p* = 0.02). The video had no significant effect in the interactive group on either the proportion extreme egalitarian (*p* = 0.52) or the proportion non‐egalitarian (*p* = 0.60). The same results were also found after excluding the five participants with nonstandard response patterns.

**Figure 2:**
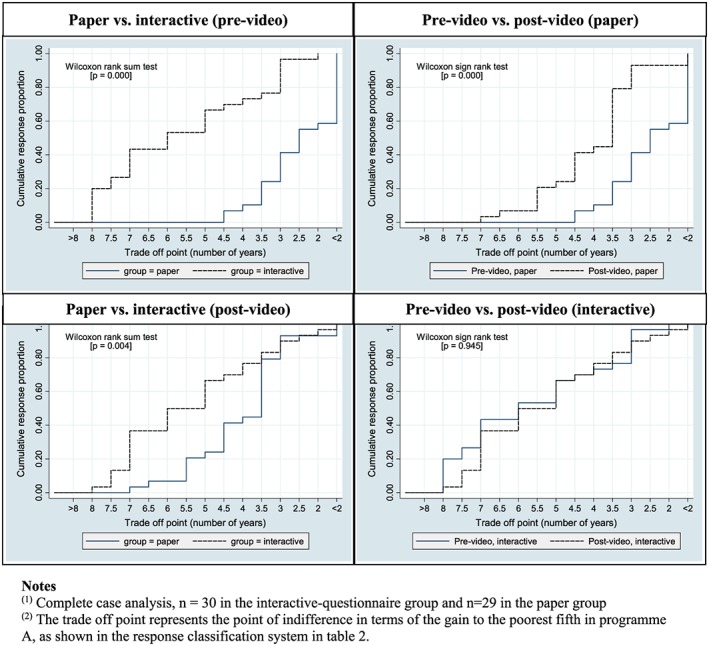
Cumulative distribution of responses [Colour figure can be viewed at wileyonlinelibrary.com]

Figure 2 shows the distribution of responses in more detail for our four comparisons. The vertical axis shows the cumulative proportion of respondents who switched to the less egalitarian Program A by that point. The stronger the inequality aversion, the later the point at which a respondent switches to A, and thus the lower the cumulative curve. The top two panels show that both e‐learning interventions have a substantial and significant effect on modifying responses away from strict egalitarianism. The bottom two panels show that the video had no significant effect on the ranking in the interactive‐questionnaire group and that the post‐video paper group had a significantly more egalitarian ranking than the post‐video interactive‐questionnaire group.

**Figure 3:**
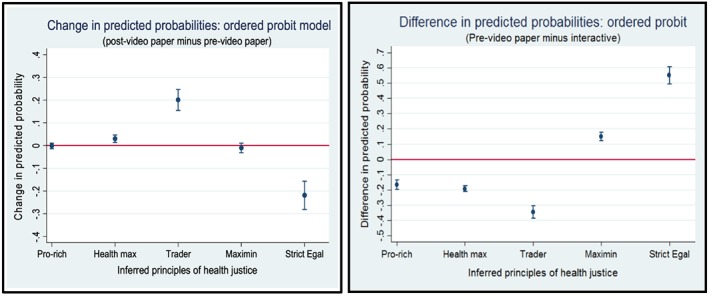
Marginal effects on probabilities, from ordered probit model with covariates [Colour figure can be viewed at wileyonlinelibrary.com]

**Figure 4:**
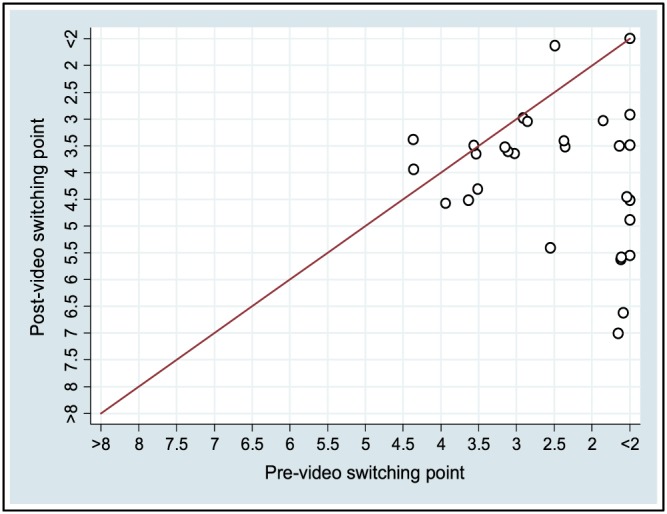
Paper group responses, pre‐ and post‐video [Colour figure can be viewed at wileyonlinelibrary.com]

Table 3 shows regression models of the five main responses categories, ordered from least to most egalitarian. The negative and significant coefficients on *interactive* (*β*_1_) confirm that the interactive questionnaire yielded less egalitarian responses than the standard paper questionnaire. The negative and significant coefficients on *post‐video* (*β*_2_) confirm that the video shifted responses in a less egalitarian direction in the paper group. Figure 3 presents these key comparisons visually, and Figure 4 shows the individual level video effects in the paper group. The joint test of (*β*_2_ + *β*_3_) shows that the video had no significant effect on responses in the interactive‐questionnaire group. Finally, the joint test of (*β*_1_ + *β*_*3*_) shows that there is weak evidence to suggest that post‐video interactive responses were less egalitarian than the post‐video paper responses. Results after excluding participants whose inconsistent responses were reinterpreted were similar (see Appendix F).

**Table 3:** Random effects ordered probit models of the five ordered response categories

Variables	Without respondent covariates	With respondent covariates
Interactive (*β*_1_)	−2.32*** (0.412)	−2.18*** (0.382)
Post‐video (*β*_2_)	−1.49*** (0.313)	−1.50*** (0.311)
Interactive * post‐video (*β*_3_)	1.70*** (0.328)	1.70*** (0.324)
Joint test of (*β*_2_ + *β*_3_): Video effect on interactive	0.21 (0.296)	0.20 (0.293)
Joint test of (*β*_1_ + *β*_3_): Interactive post‐video versus paper post‐video	−0.63* (0.356)	−0.49 (0.313)
Intercept 1 (extreme egalitarian)	−3.39*** (0.571)	−4.09*** (0.913)
Intercept 2 (maximin)	−2.70*** (0.453)	−3.43*** (0.818)
Intercept 3 (weighted prioritarian)	−0.92*** (0.286)	−1.67** (0.663)
Intercept 4 (health maximizer)	−0.23 (0.266)	−0.96 (0.635)
Observations	118	118
Number of individuals	59	59

*Note*. A positive coefficient indicates a difference in a more egalitarian direction. Standard errors in parentheses. The respondent covariates were four age groups, sex, and five deprivation quintile groups; coefficients on the covariates are suppressed as none were significant.

*** *p* < 0.01.

** *p* < 0.05.

* *p* < 0.1.

## 4 DISCUSSION

The interdisciplinary field of empirical social choice uses questionnaire methods to investigate social attitudes about distributive justice (Amiel & Cowell, 1999; Gaertner & Schokkaert, 2012). This involves asking people abstract and cognitively demanding questions about trade‐offs between competing values, such as increasing total health versus reducing health inequality. There is a risk of cognitive bias in such studies if substantial numbers of respondents only have an incomplete understanding of the question. We examined this issue in relation to one standard questionnaire method for eliciting aversion to socioeconomic health inequality. Our working hypothesis was that this questionnaire method is vulnerable to pro‐strict‐egalitarian cognitive bias in favor of responses that imply exclusive priority to reducing health inequality and unwillingness to make trade‐offs. To mitigate this potential bias, we piloted two e‐learning educational interventions designed to help respondents gain a more complete understanding of the question.

Our study adds to the large body of evidence showing that framing, priming, and other cognitive effects can be surprisingly powerful (Kahneman, 2011) and so stands as a warning to the unwary that the findings of questionnaire‐based empirical social choice studies—even ones published in prestigious academic journals—may not be quite as reliable as they appear. We found that both e‐learning interventions did indeed yield a substantially and significantly lower proportion of strict egalitarian responses (either maximin or strict egalitarian in Figure 1) than the standard questionnaire approach. In the standard paper questionnaire, just over 75% gave a strict egalitarian response implying exclusive concern to reduce health inequality rather than increase total health. In a within‐subject test, this proportion fell to 20.6% after the same subjects were exposed to our first e‐learning intervention, a video animation debate between characters advocating rival views of justice in the context of the questionnaire responses. This difference (between 75% and 20.6%) was primarily made up of respondents switching from strict egalitarian responses towards moderate egalitarian responses that do make a trade‐off between reducing health inequality and improving total health. In a randomized between‐subject test, only 23.3% gave a strict egalitarian response when exposed to our second e‐learning intervention, a spreadsheet‐based interactive version of the questionnaire providing instant feedback on the implications of alternative choices for both health inequality and total health. This difference (between 75% and 23.3%) was primarily made up of a larger proportion of respondents giving non‐egalitarian responses (either “Pro‐rich” or “Health maximizer” in Figure 1). The effects of both e‐learning interventions were highly significant in all statistical tests, including tests on the overall cumulative distribution of responses as well as the probability of a strict egalitarian response.

Importantly, however, responses still implied a high degree of health inequality aversion after the e‐learning interventions. The median trade‐off point was 5 in the interactive questionnaire groups (both pre‐ and post‐video) and 3.5 in the post‐video paper group. This implies Atkinson inequality aversion parameters of 5.4 and 10.9, respectively, implying that a marginal health gain is still valued much more highly for the poorest fifth than the richest fifth, by multiples of 2.6 and 7.0, respectively (for details of these calculations, see Robson et al., 2016).

An important strength of e‐learning interventions, as opposed to traditional face‐to‐face lectures or tutorials, is that they are consistent and auditable: All respondents are exposed to the same content, and anyone can inspect e‐learning materials and make their own judgment about how far they represent balanced education versus biasing propaganda.

An important limitation, however, is that a value judgment is required about how far the e‐learning content helps respondents gain a more complete understanding of the question, rather than merely introducing new biases. Colleagues and reviewers of this paper have identified various potential biases in our animated video. First, to help respondents remember which view was which, the extreme egalitarian character in the video was labeled a “Socialist.” Someone with centrist political views who is relaxed about large income inequalities might nevertheless be extreme egalitarian in the health domain because they think it extremely unfair for the poor to die younger than the rich—if so, they might find the label Socialist off‐putting. However, in a UK setting, the term Socialist arguably has less negative connotations than in the United States and possibly other settings, and readers need to bear in mind this cultural difference when interpreting the potential size of any such bias. Second, the character arguing for a combination view combining elements of the other views was labeled “pragmatic” and had the last and longest word. The label pragmatic itself may have positive connotations; though may also have negative connotations (e.g., lacking in principle). On the other hand, other aspects of the debate favored the extreme egalitarian character, who spoke first and had two opportunities to speak (rather than the one opportunity given to other characters).

A second limitation of this study is that we do not have information about the cognitive mechanisms through which the e‐learning interventions acted. We did not ask respondents to explain their thinking, making it hard to interpret their responses. Furthermore, we do not know whether the interventions produced their effects by helping respondents develop greater understanding of the question or by encouraging them to engage their “slow thinking” automatic cognitive systems (Kahneman, 2011), or in some other way. This may be worth investigating in future studies, by collecting information about cognitive processes. There is evidence from neuroimaging studies, for example, that “deontological” or rule‐based value judgments tend to rely on the “fast thinking” cognitive system, whereas “consequentialist” value judgments involving the weighing of outcomes require use of the slow thinking cognitive system (Greene, 2013). Future research could seek to isolate the underlying mechanisms, through experimental manipulation of the slow thinking cognitive system and the collection of data on cognitive processing.

A third limitation is that aspects of the warm up session prior to completing the questions may have primed all respondents in both paper and interactive groups to give pro‐egalitarian views. For example, although the initial presentation by the lead investigator on health inequality was balanced and factual in content and did not seek to advocate a particular ethical view, the mere fact of presenting this topic may have primed people to think about inequality as an important issue and to assume that researchers wanted them to give pro‐egalitarian responses (DeMaio, 1984). Because both groups experienced the same warm up session, the presentation was not recorded or transcribed. However, the mere fact of paying attention to the subject of inequality for a sustained period may have primed respondents to give pro‐egalitarian views, irrespective of specific details of the presentation. The e‐learning interventions may not have such large effects in other circumstances, when respondents are not primed to think about inequality as an important issue before responding to the initial questionnaire.

A fourth limitation is that the interactive spreadsheet led to a substantially higher proportion of strict non‐egalitarian responses (pro‐rich or health maximizer) than the paper questionnaire, both before and after the video intervention. After the video intervention, only one of the 29 respondents to the standard paper questionnaire (3.4%) gave a strict non‐egalitarian response (health maximizer), compared with 11 of the 30 respondents to the interactive questionnaire (four pro‐rich and seven health maximizer; 36.6% altogether). This difference was statistically significant at 10% and 5% levels respectively for full sample and subsample without inconsistent responses. One possible reason may be a form of “starting point bias” in the interactive questionnaire, because the sliding scale used for making choices was initially set at the top of the scale indicating a pro‐rich view, to align with the presentation in the paper questionnaire. This puzzling increase in strict non‐egalitarian responses suggests that our interactive spreadsheet may have introduced a new form of bias. Another issue is that “end‐point avoidance” bias in the interactive questionnaire could potentially have militated against both “Pro‐rich” and “Strict egalitarian” views, because the midpoints of the slider represented “Weighted prioritarian” views. However, (a) this speculation is inconsistent with the higher proportion of Pro‐rich views in the interactive group, and (b) switching to Program A during the midpoints of the paper questionnaire also represented Weighted prioritarian views, so it is not clear the interactive questionnaire was differentially biased in this respect.

A final issue is that value judgments about leveling‐down depend upon the reference point: Acting to harm health may seem more ethically objectionable than omitting to benefit health. Like previous studies in this field, our study provides information about the current baseline health pre‐intervention, which may act as a natural reference point. Seen from this reference point, leveling‐down in our study is naturally framed in the less objectionable sense of omitting to benefit rather than acting to harm. Removing information about current baseline health might alter the reference point in ways that encourage respondents to see leveling‐down from the more objectionable perspective of acting to harm health, which might in turn reduce the proportion of strict egalitarian responses. This hypothesis may be worth testing in future studies.

In conclusion, e‐learning interventions are not a panacea: They cannot eliminate all forms of bias, and specific features of their content may introduce new forms of bias. The values they elicit are not necessarily “better” or “closer to the truth” than those elicited through conventional methods. However, their use in this study has helped to establish that the high proportion of strict egalitarian responses often observed in previous studies may not be a reliable finding, in the sense of being stable or robust, although the extent to which e‐learning interventions would have impacted on previous estimates cannot be predicted due to difference in questionnaire design and administration. Our study suggests that e‐learning may help to avoid one specific kind of bias—that is, attributing polar extreme strict egalitarian views to respondents whose social attitudes may in fact be more nuanced. On the other hand, the interactive mode produced a number of puzzling extremely non‐egalitarian responses. Altogether, the two e‐learning interventions explored in this study have a variety of limitations, and further research is needed to develop better e‐learning interventions and explore their effects—including not only effects on responses, as explored in this study, but also effects on respondents' cognitive processes and understanding. In spite of these limitations, we believe that e‐learning interventions are a promising avenue for further research in the value elicitation field, which—carefully designed, used, and interpreted—may have a useful role to play in helping to uncover and avoid other specific forms of bias in empirical social choice studies involving trade‐offs between competing ethical values.

## CONTRIBUTION

Richard Cookson had the original idea for the study, raised the funding, led the design process, organized the experiment, and drafted the manuscript. Shehzad Ali produced the interactive questionnaire, performed the analysis, created the tables and figures, and revised the manuscript. Aki Tsuchiya and Miqdad Asaria contributed to the study design and discussion group facilitation. All four authors contributed to the study design and writing up and have approved the final manuscript.

## CONFLICTS OF INTEREST

The authors declare that they have no potential conflicts of interest, including financial, personal, or other relationships with people or organizations, that could inappropriately influence, or be perceived to influence, this study.

## STATEMENT OF THE ROLE OF THE SPONSOR

None of the sponsors in this study had any role in the study design; in the collection, analysis, and interpretation of data; in the writing of the report; or in the decision to submit the article for publication.

## STATEMENT OF RESEARCH ETHICS

Research ethics approval was obtained from the University of York Health Sciences Research Governance Committee, and informed consent was obtained from all participants.

## APPENDIX B INTERPRETATION OF NONSTANDARD RESPONSE PATTERNS IN THE PAPER GROUP

**Table:**

Original response	Decision	Justification	ID
AEBAEBA	Exclude	Clearly a misunderstanding—no classification fits.	102 (pre‐video)
BBBBBEE	Include as BBBBBEA	Clearly weighted prioritarian. Imprecision over 2 successive pairs; no precise midpoint available so err on the side of the slightly more conservative (less egalitarian) option	103 (pre‐video)
BBBEEEA	Include as BBBBAAA	Clearly weighted prioritarian. Imprecision over 3 successive pairs; take the midpoint as the switch point	117 (pre‐video)
BBBBEBB	Include as BBBBBB	Clearly extreme egalitarian. A minor and transient response error in Pair 5, corrected subsequently in Pairs 6 and 7	126 (pre‐video)
BBEEEEE	Include as BBBBBAA	Clearly weighted prioritarian. Imprecision over 5 successive pairs; choose the midpoint as the switch point	101 (post‐video)
BEEAAAA	Include as BEAAAAA	Clearly health maximizer. Imprecision over 2 successive pairs; no precise midpoint available so err on the side of the slightly more conservative (less egalitarian) option	127 (post‐video)
AAEBBEA	Exclude	Clearly a misunderstanding—no classification fits.	102 (post‐video)
BBBBEEA	Include as BBBBEAA	Clearly weighted prioritarian. Imprecision over 2 successive pairs, no precise midpoint available so err on the side of the slightly more conservative (less egalitarian) option	117 (post‐video)
BBBEEAA	Include as BBBEAAA	Clearly weighted prioritarian. Imprecision over 2 successive pairs, no precise midpoint available so err on the side of the slightly more conservative (less egalitarian) option	126 (post‐video)

## APPENDIX D VIDEO ANIMATION TRANSCRIPT

Narrator: Now that you've seen our question about health inequality trade‐offs, how will you respond? It's a tricky question to answer, because it raises an ethical dilemma. When you start to think about it, you find yourself torn between competing ethical principles. Philosophers have argued about these principles for thousands of years, so it's not surprising that reasonable people can disagree and give different answers. This short animation is designed to help you think through the different arguments and make up your own mind. We have four characters, with different views about what answers to give. Let's start with Simon the socialist. Simon—over to you.
Simon the Socialist: Hi! I'm Simon the Socialist. Health inequality is unjust, because it blights the lives of millions of people. I find it shocking that in England today, rich people live 12 more years of life in full health than poor people. That's simply not acceptable. We should do everything we can to reduce these unjust inequalities in health. That's why I will always choose Program B—because it gives more years to the poor than the rich and so reduces health inequality. I will NEVER choose Program A.
Narrator: Thanks Simon. Now let's have an opposing view from Emma the Economist.
Emma the Economist: Hi! I'm Emma the Economist. I can see Simon's point, though don't feel so strongly. I do have a strong view, however, about which options you should definitely NOT choose. If the poor get fewer years in Program B, this can actually make them worse off. I think you should avoid choosing this “lose–lose” option that makes everyone worse off.
Simon the Socialist: Yes but this is still fairer because it gives the rich fewer years than the poor. It therefore still reduces inequality.
Emma the Economist: I disagree. How can it be fair to choose an option that makes the poor worse off? That's not fair—it's bad for everyone! If Simon is concerned about the poor, he should not choose an option that gives them worse health. In fact, he shouldn't even choose an option that gives the poor the same health and the rich worse health. A “lose–same” option of this kind is still an unnecessary waste of human life among the rich. So in both lose–lose and lose–same cases, I would choose Program A. But I don't have a strong view about what to do in the other, “win–win,” options.
Narrator: Thanks Emma and Simon. Now let's hear from Harry the health maximizer.
Harry the Health Maximizer: Hi! I'm Harry the health maximizer. I agree with Emma the Economist, but I would take her argument further. What matters is improving people's health. Everyone's health matters equally—improving rich people's health is no more and no less important than improving poor people's health. So we should simply try to improve total health as much as we can. That's why I will almost always choose Program A—because Program A almost always delivers more total health than Program B. I will just add up the total health under each option and choose the one with the higher total. Where both options result in the same total health, I would say they are equally good.
Narrator: Thanks Harry. Now let's hear from Priya the pragmatist.
Priya the Pragmatist: Hi! I'm Priya the Pragmatist. I agree with Simon the Socialist that reducing inequality is important. I also agree with Emma the Economist that we should not waste human life. And I also agree with Harry the Health Maximizer that improving total health matters. They all make good points. But they all take things to extremes—they only see one side of the story. I suggest a sensible, pragmatic solution. Let's try to reduce inequality but without losing too much total population health and without choosing lose–lose options. So we give some weight to reducing health inequality, as Simon wants, some weight to avoiding lose–lose options, as Emma wants, and some weight to improving total population health, as Harry wants. At one extreme, Simon will always choose Program B. And at the other extreme, Harry will almost always choose Program A. I will choose something in between. I will choose Program B to start with, in order to reduce health inequality. But then at some point, I will switch to Program A when the total health gain from Program B becomes too small. I will switch because I care about improving total population health as well as reducing health inequality. And I will switch BEFORE Program B becomes a lose–same or lose–lose option, because I accept the point make by Emma the Economist. I'm a pragmatist, so I want to weigh up all of those competing considerations and take a balanced compromise approach.
Narrator: Thanks Priya! Ok, there we have it—four different views. Now its up to you to decide. Do you agree with Simon the Socialist, who would always choose Program B because it reduces health inequality? Or Emma the Economist, who would never choose Program B when it's a lose–lose or lose–same option. Or Harry the Health Maximizer, who would almost always choose Program A because it gives the greatest total improvement in population health? Or finally, do you agree with Priya the Pragmatist, who will start by choosing Program B and then switch at some point to Program A, because she wants to find a compromise approach that gives some weight to all these conflicting considerations. The choice is yours!

## APPENDIX F SENSITIVITY ANALYSIS: RANDOM EFFECTS ORDERED PROBIT MODELS OF THE FIVE ORDERED RESPONSE CATEGORIES AFTER EXCLUDING FIVE RESPONDENTS WITH INCONSISTENT RESPONSES

**Table:**

Variables	Without respondent covariates	With respondent covariates
Interactive (*β*_1_)	−2.33*** (0.442)	−2.25*** (0.411)
Post‐video (*β*_2_)	−1.37*** (0.325)	−1.37*** (0.325)
Interactive × post‐video (*β*_3_)	1.58*** (0.342)	1.58*** (0.339)
Joint test of (*β*_2_ + *β*_3_): Video effect on interactive	0.21 (0.296)	0.20 (0.294)
Joint test of (*β*_1_ + *β*_3_): Interactive post‐video versus paper post‐video	−0.75** (0.381)	−0.67** (0.341)
Intercept 1 (extreme egalitarian)	−3.39*** (0.619)	−4.04*** (1.029)
Intercept 2 (maximin)	−2.71*** (0.494)	−3.38*** (0.930)
Intercept 3 (weighted prioritarian)	−0.93*** (0.319)	−1.62** (0.763)
Intercept 4 (health maximizer)	−0.21 (0.299)	−0.88 (0.725)

*Note*. A positive coefficient indicates a difference in a more egalitarian direction. Standard errors in parentheses. The respondent covariates were four age groups, sex, and five deprivation quintile groups; coefficients on the covariates are suppressed as none were significant.

*** *p* < 0.01.

** *p* < 0.05.

* *p* < 0.1.
